# Supplementary material for: Individualized PEEP without Recruitment Maneuvers Improves Intraoperative Oxygenation: A Randomized Controlled Study
Source: Bioengineering (Basel). 2023 Oct 9;10(10):1172. doi: 10.3390/bioengineering10101172 (PMC10604161; doi:10.3390/bioengineering10101172)
Supplement: Supplementary file 1 [file bioengineering-10-01172-s001.zip › bioengineering-2559062-supplementary.pdf]

Supplementary Figure S1 Experimental protocol and interventions.

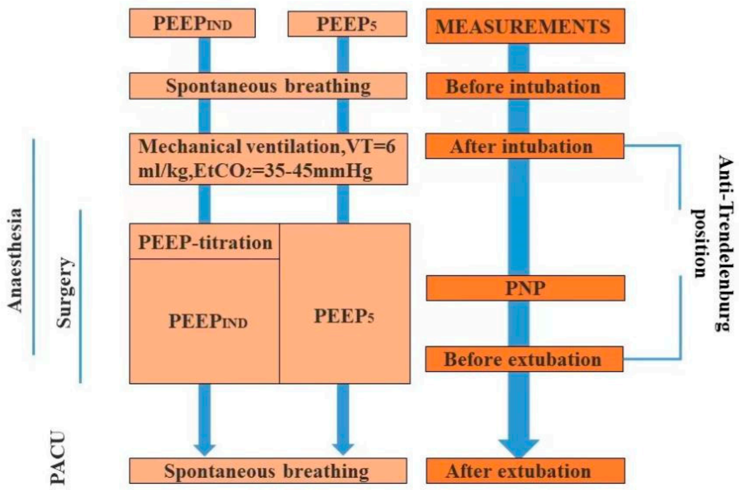

PACU, post-anaesthesia care unit; $PEEP_{IND}$, individualized PEEP setting guided by electrical impedance tomography; $PEEP_5$, standard PEEP setting of 5 cmH_2O; PNP, pneumoperitoneum.
